# Mesenchymal stem cells in the treatment of Cesarean section skin scars: study protocol for a randomized, controlled trial

**DOI:** 10.1186/s13063-018-2478-x

**Published:** 2018-03-02

**Authors:** Dazhi Fan, Qing Xia, Shuzhen Wu, Shaoxin Ye, Li Liu, Wen Wang, Xiaoling Guo, Zhengping Liu

**Affiliations:** 1 Department of Obstetrics, Southern Medical University Affiliated Maternal & Child Health Hospital of Foshan, 11 Renminxi Road, Foshan, Guangdong 528000 China; 2 Foshan Institute of Fetal Medicine, Southern Medical University Affiliated Maternal & Child Health Hospital of Foshan, Foshan, Guangdong 528000 China; 3 Department of Epidemiology and Biostatistics, School of Public Health, Anhui Medical University, Hefei, Anhui 230032 China; 4 Menzies Institute for Medical Research, University of Tasmania, Private Bag 23, Hobart, Tasmania 7000 Australia; 5 First Affiliated Hospital, College of Medicine, Zhejiang University, Hangzhou, Zhejiang 310003 China

**Keywords:** Mesenchymal stem cells, Cesarean section skin scars, Randomized controlled trial, Protocol

## Abstract

**Background:**

Cesarean delivery has already become a very common method of delivery around the world, especially in low-income countries. Hypertrophic scars and wound infections have affected younger mothers and frustrated obstetricians for a long time. Mesenchymal stem cells (MSCs) have strong potential for self-renewal and differentiation to multilineage cells. Previous studies have demonstrated that MSCs are involved in enhancing diabetic wound healing. Therefore, this study is designed to investigate the safety and efficacy of using MSCs in the treatment of Cesarean section skin scars.

**Methods:**

This trial is a prospective, randomized, double-blind, placebo-controlled, single-center trial with three parallel groups. Ninety eligible participants will be randomly allocated to placebo, low-dose (transdermal hydrogel MSCs; 3 × 10^6^ cells) or high-dose (transdermal hydrogel MSCs; 6 × 10^6^ cells) groups at a 1:1:1 allocation ratio according to a randomization list, once a day for six consecutive days. Study duration will last for 6 months, comprising a 1 week run-in period and 24 weeks of follow-up. The primary aim of this trial is to compare the difference in Vancouver Scar Scale rating among the three groups at the 6th month. Adverse events, including severe and slight signs or symptoms, will be documented in case report forms. The study will be conducted at the Department of Obstetric of Southern Medical University Affiliated Maternal & Child Health Hospital of Foshan.

**Discussion:**

This trial is the first investigation of the potential for therapeutic use of MSCs for the management of women’s skin scar after Cesarean delivery. The results will give us an effective therapeutic strategy to combat Cesarean section skin scars, even with uterine scarring.

**Trial registration:**

ClinicalTrials.gov, NCT02772289. Registered on 10 May 2016.

**Electronic supplementary material:**

The online version of this article (10.1186/s13063-018-2478-x) contains supplementary material, which is available to authorized users.

## Background

Over the past decades, Cesarean delivery has already become a very common method of delivery around the world, especially in low-income countries [1]. In China, although the Cesarean rate has decreased in individual megacities, the overall annual rate has still increased, reaching 34.9% in 2014, from 28.8% in 2008 [2]. Hypertrophic scars and wound infections have affected younger mothers and frustrated obstetricians for a long time. As one of the top three hospital-acquired infections, wound infections can prolong hospitalization and greatly increase the rates of hospital readmission, risk of death, and overall costs of healthcare [3]. It is reported that the incidence of wound infections ranges from 3% to 20% in women after Cesarean delivery [4]. In addition, hypertrophic scars and psychological stress are causes of dissatisfaction in women after Cesarean delivery.

The cutaneous wound healing process is very complex. It requires a variety of cells to collaborate, such as resident cells of the skin, hematopoietic cells, and immune cells [5]. Many complex factors, such as abnormal macrophage polarization, abnormal keratinocyte and fibroblast migration, proliferation, differentiation and apoptosis, impaired recruitment of mesenchymal stem cells (MSCs) and endothelial progenitor cells, and decreased vascularization may contribute to an abnormal wound healing process [6–8]. It is also reported that enhanced and prolonged expression of tumor necrosis factor-alpha contributes to abnormal wound healing processes [9, 10].

Hypertrophic scarring is a fibrotic disease, arising from fibroproliferation disorder, which occurs after the damage of the deep dermis by deep skin injury, surgical procedure, or burns [11]. Overproduction of extracellular matrix and collagens are considered to be the main pathological characteristics of hypertrophic scars [12, 13]. Key cell subpopulations, including deep dermal fibroblasts, myofibroblasts, fibrocytes, and T-helper cells, could both modify and interact with the extracellular matrix of the wound, ultimately forming a hypertrophic scar [14]. Abnormal wound healing processes can also cause bacterial proliferation. In some instances, the bacterial load increases sufficiently for infection to ensue [15]. Therefore, an abnormal wound healing process could increase the risk of hypertrophic scarring and wound infection. In turn, continuous inflammation and bacterial proliferation also increase the risk of abnormal wound healing processes.

Post-Cesarean wound infection has been ascribed to many factors, including obstetrician-related factors, such as skin incision type, time of operation, suture technique, or intraoperative blood loss, and maternal factors, such as intrauterine infection prior to delivery, presence of comorbidities, or body mass index [16, 17]. Antibiotics, wound exploration, and debridement are mainstays in the medical care of post-Cesarean wound infections at present [18]. Many therapeutic approaches, such as laser therapy, radiation, cryotherapy, cryosurgery, or intralesional injections of corticosteroids, have also been reported in the management of hypertrophic scars [19]. However, many of them are involved in high rates of recurrence, and many are also expensive and painful.

Mesenchymal stem cells are a population of pluripotent stem cells. They possess high potencies of self-renewal and differentiation into canonical cells of the mesenchyme [20]. They are initially discovered in bone marrow and are subsequently found in almost every type of tissue, including the endometrium, placenta, umbilical cord, adipose tissue, and gingiva [21]. They have been reported as an effective and successful treatment for many diseases, including rheumatoid arthritis [22], experimental autoimmune encephalomyelitis [23], bone regeneration [24], and ischemic cardiomyopathy [25]. As a treatment modality, MSCs have demonstrated great potential value.

Through their migratory, anti-inflammatory, and trophic properties, MSCs exert numerous functions that may be of relevance for restoring skin tissue function and enhancing healing [26]. Using a rabbit model, researchers found that human MSCs can regulate inflammation and prevent the formation of hypertrophic scars [27]. Previous results of clinical trials also demonstrated the benefits derived by the employment of MSCs in wound healing [28, 29]. Falanga *et al.* [30] indicated that MSCs can be safely and effectively delivered to wounds using a fibrin spray system. Another clinical trial showed that directly applied bone marrow-derived cells can lead to dermal rebuilding and closure of nonhealing chronic wounds [31]. Yoshikawa *et al.* [32] demonstrated that MSCs are therapeutically effective in patients with intractable dermatopathies. In addition, Dash *et al.* [33] showed that autologous implantation of bone marrow-derived MSCs in nonhealing ulcers accelerated the healing process and significantly improved clinical parameters.

In this trial, we hypothesize that MSCs can reduce hypertrophic scars and decrease wound infection after Cesarean delivery. Therefore, we undertake a Phase II clinical trial to evaluate the safety and efficacy of MSCs in the treatment of Cesarean section skin scars in a prospective, randomized, double-blind, placebo-controlled, single-center study.

## Methods

### Study design

**Fig. 1:**
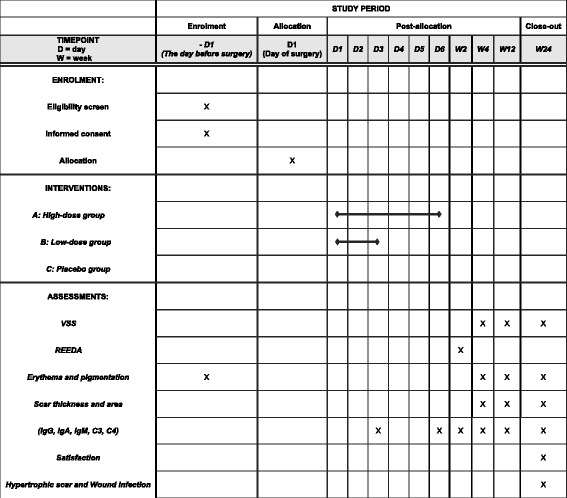
SPIRIT figure

**Fig. 2:**
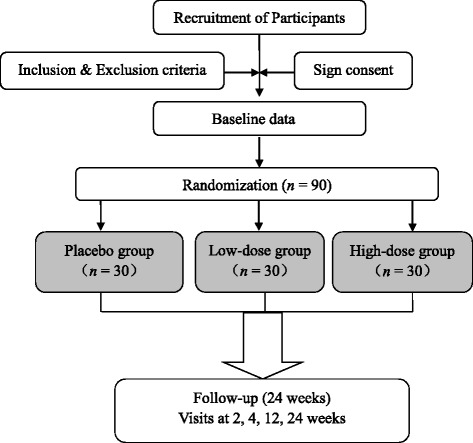
Trial schema

This study protocol conforms to the Standard Protocol Items: Recommendations for Interventional Trials (SPIRIT) guidelines (see Fig. 1 and Additional file 1). The trial is intended to target primiparous women between the 37th and 42nd weeks of gestation. Trained out-patient doctors will introduce the details of the trial to each potential participant during a clinic visit. The trial coordinator will contact the interested participant by mobile phone and WeChat, a very popular social networking app in China [34]. Eligible participants will be introduced in the trial. The inclusion and exclusion criteria and trial flow are shown in Table 1 and Fig. 2, respectively. The selection, information process, and randomization will be implemented as soon as we know that the primiparous woman is going to have a programmed Cesarean delivery. There is about 1 day between selection and delivery. The Cesarean delivery will be programmed for a gestational age ≥ 37 weeks and < 42 weeks. This trial will be a prospective, randomized, double-blind, placebo-controlled with three parallel groups. The trial will be conducted at the Department of Obstetric of Southern Medical University Affiliated Maternal & Child Health Hospital of Foshan.

**Table 1:** Eligibility criteria

Inclusion criteria	Exclusion criteria
Primiparous women receiving Cesarean delivery	Any systemic uncontrolled disease
Aged 21–35 years	Recent or current cancer
Gestation age ≥ 37 weeks and < 42 weeks	History of or presenting with a keloid formation
Willing to sign an informed consent form and a photographic release form	Wounds or local disease in treatment area
Willing to comply with study dosing and complete entire course of study	Planning any other cosmetic procedure to the study area during the study period
	Smoking

### Interventions

Eligible participants, who have signed informed consent forms, will be randomized to placebo, low-dose (3 × 10^6^ cells) or high-dose (6 × 10^6^ cells) groups, receiving transdermal hydrogel MSCs or placebo once a day for six consecutive days. Each pump dispenses 1.0 ml gel (including 1 × 10^6^ cells or none). The justification of doses is based on previous studies for other indications [35, 36] and our preliminary experiment (unpublished). Each pump, either MSCs or placebo, has the same external characteristics except for a digital tag, to allow blinding of the intervention. After suturing the skin incision as usual, the first intervention will take place on the operating table and the remaining five interventions will take place in the postnatal ward. Participants in the placebo group will receive placebo hydrogel once a day for 6 days; those in the low-dose group will receive one dose of hydrogel with cells once a day for three consecutive days and then placebo hydrogel for the next three consecutive days; those in the high-dose group will receive cells for 6 days.

The government has been committed to promoting cooperation between governments, academia, pharmaceutical companies, biotechnology firms, and private investors to research and evaluate MSC therapies, since they are advanced therapies [37, 38]. The Health-Biotech Pharmaceutical Company (Beijing, China) manufactures both MSCs and the placebo hydrogel. The products will be provided without charge. The MSCs are extracted from the umbilical cord, which is donated by a healthy donor who has provided informed consent. Detailed descriptions of the method have been given in a previous article [39].

The study duration will last for 6 months, including a 1 week run-in period and 6 months of follow-up. All participants will be assessed at 0.5, 1, 3, and 6 months during the follow-up period. Table 2 provides the time points and specific measurements of data. Cosmetic use may affect wound function. If participants use cosmetics in the follow-up period, they will be withdrawn from the study.

**Table 2:** Schedule of visits and assessments

Time point (weeks)	2	4	12	24
Vancouver Scar Scale		√	√	√
REEDA scale	√			
Erythema		√	√	√
Pigmentation		√	√	√
Scar thickness		√	√	√
Scar area		√	√	√
Immunoglobulin		√	√	√
Satisfaction		√	√	√
Adverse events	√	√	√	√

REEDA, Redness, Edema, Ecchymosis, Discharge, Approximation

### Outcomes

#### Primary outcome

The primary aim of this study is to compare the difference in Vancouver Scar Scale (VSS) rating among the three groups in the 6th month. The VSS rates vascularity (normal, pink, red, or purple), pigmentation (normal, hypopigmented, mixed, or hyperpigmented), height (flat, < 2 mm, 2–5 mm, or > 5 mm), and pliability (normal, supple, yielding, firm, ropes, or contracture). The Chinese version of the VSS has been shown to have good intraclass correlations and Cronbach’s *α* measures [40, 41]. All scars will be assessed independently by two observers (SW and SY) on the same day when the participants are lying in a supine position with the scar exposed in bright light. If the data varies, another researcher (DF) will be required to assess the scar at the same day and the results with the highest frequency will be recorded.

#### Secondary outcomes

The secondary outcome measures are as follows:

1. The VSS at 1 and 3 months after treatment
  The VSS will be also evaluated at 1 and 3 months after treatment. The differences in the 1st and 3rd month will also be compared among the three groups.
2. Wound healing
  Wound healing status will be assessed 14 days after surgery using the REEDA scale. The REEDA scale contains five variables: redness, edema, ecchymosis, discharge, and the approximation of wound edges [19].
3. Erythema and pigmentation
  These will be measured using a narrowband reflectance spectrophotometer (Mexameter MX18) at 1, 3, and 6 months after treatment.
4. Scar thickness and area
  The scar thickness and area will be measured using a high definition ultrasound device at 1, 3, and 6 months after treatment.
5. Meanwhile, the mother’s milk will be collected at each visit and immunoglobulins (IgG, IgA, IgM) and complements (C3, C4) will be detected by the transmission immune turbidity method using an automatic biochemical analyzer.
6. Participants’ satisfaction of the treatment will be measured using a satisfaction scale, including five categories, namely, none, slight, moderate, good, and very good.
7. The number of hypertrophic scars and wound infections during the 6 months.
8. Safety and tolerability of the intervention.

All adverse events, including side effects and other ailments, will be recorded in a case report form. The researcher will report all adverse events to the ethics committee. For all participants, additional services arising from all adverse events will be provided free of charge.

### Sample size

Our sample size calculation is based on previous results from Professor Yu-Chen Huang’s preliminary trial in Taipei Medical University WanFang Hospital [42]. These results found that the mean VSS was 4.50 ± 1.68 in healthy pregnant women. The ratio of the MSC and control group sample size sets was 2:1. We will use the conventional values *α* = 0.05 and *β* = 0.1 for two-sided tests of probability. Meanwhile, we hypothesize that the difference in mean VSS at 6 months after treatment between the MSCs and placebo group will be *δ* = 1.5. The total sample size is estimated to be 74. Considering a dropout and other potential influencing factors, the final participant is estimated to be about 90 (*n* = 30 in each group).

### Randomization, concealment, and blinding

A computerized random number generator will be used to produce a randomization schedule employing simple randomization by an independent clinical researcher, who is not involved in the recruitment, intervention, assessment, or statistical analysis. During the study period, the independent clinical researcher will retain the randomization list. The randomization sequences will be concealed in lightproof, sealed envelopes. After signing informed consent forms, eligible participants will be randomly allocated to the three parallel groups at a 1:1:1 allocation ratio, according to the randomization list.

Each participant will receive a unique randomized number. Meanwhile, each participant’s pump will be labeled with a unique randomization number, which will become the participant’s number. Direct participants and investigators, including the outcome assessors and statisticians, will be blinded to the allocation status throughout the study. Once participants have been allotted randomized numbers, a reasonable effort will be made by investigators to avoid missing data.

### Data collection and management

All research investigators will be trained uniformly in standard operating procedures. A regular monitoring scheme will be set up to collect accurate and valid data. Non-numeric data will be converted into numbers for storage. All laboratory specimens will be identified only by a coded identification number to maintain participant confidentiality. To ensure confidentiality, data dispersed to project team members will be blinded to any identifying participant information. After verification of the content, two research investigators will independently input the data into a database. All participant information will be kept safely under confidential conditions and archived for 10 years. The project principal investigator could access the full data. Participant recruitment is currently in progress. The first participant was recruited on 14 September 2016.

Meanwhile, a data monitoring committee will be established, which will be independent of the study organizers and will periodically review the accumulating data and determine whether the trial should be modified or discontinued. The committee members will perform independent review of trial processes every 2 months.

### Statistical analysis

All analyses will be conducted on the intention-to-treat and per-protocol principles. Regardless of whether participants received the randomized treatment, the intention-to-treat principle considers all of them as randomized. A complete case analysis will be performed if missing data for the randomized participants accounts for less than 5% of total data. Multiple imputations will be used if missing data is more than 5%. Dropouts will be included in the analysis by multiple imputations for missing data. The effect that the per-protocol participants and missing data might have on results will be assessed via sensitivity analysis. Subgroup analysis will be performed based on age and gestational age.

The primary comparison, both doses vs. placebo, will be analyzed using a superiority analysis. We will use the *t* test for the continuous outcome. Meanwhile, high dose vs. placebo and low dose vs. placebo will also be analyzed using the *t* test. Other outcomes will be analyzed using noninferiority analysis. The mean (and standard deviation) will be expressed for continuous variables, and numbers (percentages) will be expressed for categorical variables. Group variances will be compared using Leven’s test at the 0.05 significance level. Analysis of variance (ANOVA) will be used to analyze continuous data, and the *chi*-square test or Fisher’s exact test will be used for categorical data. To investigate the effects of treatment and time course, repeated measures ANOVA will be applied to determine changes in the continuous outcome data at each visit. R 3.1 software will be used for analysis.

## Discussion

Mesenchymal stem cells have a strong potential for self-renewal and differentiation to multilineage cells. They can secrete several extracellular matrix molecules, growth factors, and cytokines that play a pivotal role in the regulation of angiogenesis and immune and inflammatory responses [19, 43]. Currently, several clinical trials have found potential value in using MSCs in healing chronic and acute wounds and scar remodeling [44].

To assess the efficacy and feasibility of autologous bone marrow-derived MSCs in the treatment of chronic nonhealing ulcers, researchers designed a randomized control study on a series of 24 participants with a history of nonhealing leg ulcer [33]. After 12 weeks, compared with control participants, the treatment participants had significant improvement in reduction in ulcer size. These results indicated that autologous implantation of bone marrow-derived MSCs in nonhealing ulcers accelerated the healing process. A single-arm clinical trial also indicated that autologous MSCs were shown to be therapeutically effective in patients with skin wounds [32]. To identify better cells for the treatment of diabetic foot ulcers, a randomized controlled trial was conducted on a sample of 41 type 2 diabetic patients with bilateral foot ulcer [45]. All patients were injected intramuscularly with bone marrow MSCs, bone marrow-derived mononuclear cells, or normal saline as placebo. They found that bone marrow mesenchymal stem cell therapy might be more effective than bone marrow-derived mononuclear cell therapy in promoting foot ulcer healing in diabetic patients. Another similar randomized controlled trial also obtained a similar result [46]. The frequency of major limb amputation was lower in a group treated with autologous bone marrow stem cells than in a group receiving standard medical care. Meanwhile, a meta-analysis demonstrated that autologous stem cell transplantation can be considered a safe and effective approach for treatment of many patients with diabetes mellitus [47].

Although previous results of trials with MSCs in wound healing have been reported, the application of MSCs on Cesarean section skin scars has not yet been investigated. In this prospective, randomized, double-blind, placebo-controlled study, we aim to investigate the possible effects of MSCs in women’s skin scars after Cesarean delivery. We hypothesize that MSCs can enhance wound healing, reduce hypertrophic skin scars, and decrease wound infection. To our knowledge, this trial is the first to investigate the potential of the therapeutic use of MSCs for the management of women’s skin scars after Cesarean delivery. The outcomes from this trial will help to determine the efficacy and safety of MSC treatment in Cesarean section skin scars. The results will also identify a therapeutically effective dose of MSCs in preventing hypertrophic scars and wound infections risk factors. It will give us an effective therapeutic strategy to combat Cesarean section skin scars, even with uterine scarring.

A limitation of this trial is that this is a single-center clinical trial, which will limit the extrapolation of results. Meanwhile, loss of participants at follow-up is possible, especially for nonresponders in the prospective trial study. Notwithstanding its limitations, this trial will suggest whether MSC can be a safe and effective in the treatment of Cesarean section skin scars.

## Trial status

Participant recruitment is currently in progress. The first participant was recruited on 14 September 2016. We hope to complete enrolment for the trial by March 2018 with all 6 month follow-up data expected by September 2018.

## Additional file

Additional file 1: SPIRIT checklist. (DOCX 43 kb)

## Abbreviations

ANOVA: Analysis of variance
MSCs: Mesenchymal stem cells
REEDA: Redness, Edema, Ecchymosis, Discharge, and Approximation
VSS: Vancouver Scar Scale
